# Energetic determinants of animal cell polarity regulator Par-3 interaction with the Par complex

**DOI:** 10.1016/j.jbc.2022.102223

**Published:** 2022-07-01

**Authors:** Rhiannon R. Penkert, Elizabeth Vargas, Kenneth E. Prehoda

**Affiliations:** Department of Chemistry and Biochemistry, Institute of Molecular Biology, University of Oregon, Eugene, Oregon, USA

**Keywords:** cell polarity, protein interactions, protein kinase, pdz domains, protein reconstitution, aPKC, atypical Protein Kinase C, APM, aPKC phosphorylation motif, BR, basic region, PBM, PDZ-binding motif

## Abstract

The animal cell polarity regulator Par-3 recruits the Par complex (consisting of Par-6 and atypical PKC, aPKC) to specific sites on the cell membrane. Although numerous physical interactions have been reported between Par-3 and the Par complex, it is unclear how each of these interactions contributes to the overall binding. Using a purified, intact Par complex and a quantitative binding assay, here, we found that the energy required for this interaction is provided by the second and third PDZ protein interaction domains of Par-3. We show that both Par-3 PDZ domains bind to the PDZ-binding motif of aPKC in the Par complex, with additional binding energy contributed from the adjacent catalytic domain of aPKC. In addition to highlighting the role of Par-3 PDZ domain interactions with the aPKC kinase domain and PDZ-binding motif in stabilizing Par-3–Par complex assembly, our results indicate that each Par-3 molecule can potentially recruit two Par complexes to the membrane during cell polarization. These results provide new insights into the energetic determinants and structural stoichiometry of the Par-3–Par complex assembly.

The Par complex polarizes diverse animal cells by forming a specific domain on the plasma membrane. In the Par domain, the Par complex component atypical PKC (aPKC) phosphorylates and displaces substrates, thereby restricting them to a complementary membrane domain (1). In this manner, the cellular pattern formed by Par-mediated polarity is ultimately determined by the mechanisms that target the Par complex to the membrane. Membrane recruitment relies at least in part on interactions with proteins that directly associate with the membrane, including the Rho GTPase Cdc42 and the multi-PDZ protein Par-3. The Par complex’s interaction with Cdc42 is *via* a single well-defined site, the Par complex component Par-6’s semi-CRIB domain (2, 3, 4, 5, 6). However, numerous interactions between Par-3 and the Par complex have been reported (2, 3, 7, 8, 9, 10) and it has been unclear how each contributes to the overall interaction.

The interaction between Par-3 and the Par complex was originally discovered in the context of the interaction between aPKC and its phosphorylation site on Par-3, the aPKC phosphorylation motif (APM, aka Conserved Region 3–CR3) (7). Subsequently, the following interactions were reported outside of aPKC’s catalytic domain: (i) Par-3 PDZ1 and the Par-6 PDZ (2, 3, 11), (ii) Par-3 PDZ2-3 acting together and aPKC (8), (iii) Par-3 PDZ1 or PDZ3 binding to the Par-6 PDZ-binding motif (PBM) (9), and (iv) PDZ2 with a PBM in aPKC (10) (Fig. 1*A*). Each of these interactions, except for the interaction of aPKC’s kinase domain with its substrate sequence on Par-3, involves one or more of Par-3’s three PDZ protein interaction domains.

**Figure 1:**
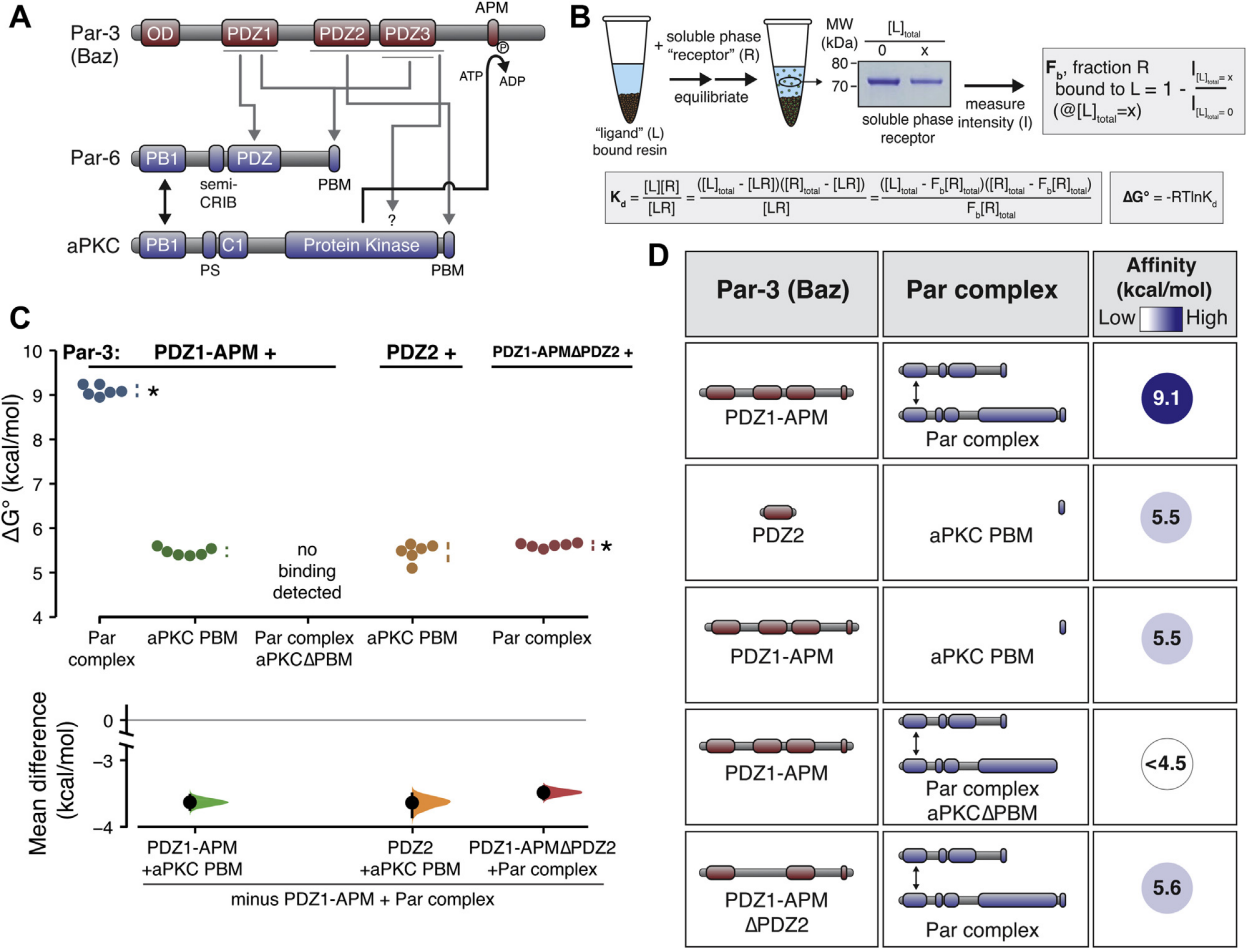
**Energetic composition of the Par-3 interaction with the Par complex.***A*, a schematic of reported Par-3 interactions with the Par complex. *B*, a schematic of the supernatant depletion quantitative binding assay and key equations used to calculate the fraction of “R” bound to “L” (F_b_), the equilibrium dissociation binding constant (K_d_), and ultimately the binding energy (ΔG°). *C*, cumming estimation plot of Par-3–Par complex interaction energies measured using the supernatant depletion assay. The result of each replicate is shown (*filled circles*) along with mean and SD (*gap* and *bars* adjacent to *filled circles*) are shown in the *top plot*. The difference in the means relative to the PDZ1-APM–Par complex mean is shown in the *bottom plot* (*filled circles*), along with the 95% confidence intervals (*black bars*) derived from the bootstrap 95% confidence interval (*shaded distribution*). *Asterisks* indicate apparent values that may be the result of multiple binding interactions. *D*, the summary of binding energies for Par-3 and Par complex variants. APM, aPKC phosphorylation motif.

Several factors have made it difficult to understand how these many interactions identified between Par-3 and the Par complex contribute to the overall interaction. Most interactions have not been examined in the context of the intact Par complex. In this context, it is not possible to understand how individual interactions contribute to the overall energetics of Par-3 assembly with the Par complex or if interactions might cooperate or compete. Furthermore, many of the interactions have not been examined quantitatively, so it has not been possible to assess their relative strength. Finally, the presence of multiple potential Par complex–binding sites on Par-3 raises the possibility that each Par-3 protein might bind more than one Par complex. Here, we examine the energetics of Par-3 binding to the fully reconstituted Par complex using a quantitative binding assay to address these issues.

## Results

### Multiple interactions contribute to Par-3–Par complex interaction energy

To investigate the energetic determinants of Par-3’s interaction with the Par complex (Par-6 and aPKC), we measured binding energy using a supernatant depletion assay (Fig. 1*B*), using the *Drosophila* proteins. The supernatant depletion assay uses solid (glutathione or amylose agarose resin) and soluble phases like a typical “pull-down” assay but the amount of protein in the soluble phase (“receptor”) is monitored at the equilibrium rather than what remains in the solid phase after washing (12) (Fig. 1*B* and S1*A*). To confirm that the supernatant depletion assay yields similar affinities to another established protein interaction assay, we measured the affinity of the Crumbs intracellular domain for the Par-6 CRIB-PDZ (6.89 ± 0.07 kcal/mol; mean ± 1 SD, n = 6). This result is indistinguishable from measurements made using the fluorescence anisotropy method (6.89 ± 0.06 kcal/mol) (13). For measuring Par complex affinities for Par-3, we used the PDZ1-APM region of Par-3 (Fig. 1*A*) as a starting point because it contains all domains that have been reported to interact with the Par complex and it can be purified to a level suitable for quantitative analysis (all protein reagents used in this study are shown in Fig. S1*B*) (10). We examined the binding of Par-3 PDZ1-APM to the full Par complex to allow the multiple, potentially cooperative interactions to form. As shown in Fig. 1, *C* and *D*, the binding energy (ΔG°) of Par-3 PDZ1-APM to the Par complex is 9.1 kcal/mol (9.0–9.2 95% CI; all binding energies reported in this study can be found in Table S1). Because of the potential for multiple interactions between Par-3 and the Par complex, this energy may be the cumulative effect of individual binding events. For this reason, binding energies for reactions that have stoichiometries that are potentially greater than one are labeled as “apparent”. Below, we examine how each of the potential interactions between Par-3 and the Par complex contributes to the overall binding energy.

We recently discovered an interaction between the second of Par-3’s three PDZ domains (PDZ2) and a highly conserved PBM at the COOH-terminus of aPKC (10). The Par-3 PDZ2–aPKC PBM interaction is required for the recruitment of the Par complex to the cortex of asymmetrically dividing *Drosophila* neural stem cells. Using the supernatant depletion assay, we found that this interaction has an apparent binding energy of 5.5 kcal/mol (5.3–5.7 95% CI), which represents approximately 60% of the full Par-3 PDZ1-APM’s binding energy and is indistinguishable from PDZ1-APM binding to the aPKC PBM (Fig. 1, *C* and *D*). We were unable to detect an interaction between PDZ2 and a Par complex lacking aPKC’s PBM (the limit of detection of the supernatant depletion assay is approximately 4.5 kcal/mol) consistent with a central role for this motif in the overall interaction. Surprisingly, however, removal of PDZ2 did not abrogate binding as PDZ1-APMΔPDZ2 bound the Par complex with approximately the same binding energy as that for Par-3 PDZ2–aPKC PBM (Fig. 1, *C* and *D*; 5.6 kcal/mol; 5.6–5.7 95% CI). We conclude that while Par-3 PDZ2–aPKC PBM represents a significant fraction of the Par-3 interaction with the Par complex, interactions outside of the PDZ2 (but also potentially involving the aPKC PBM) make a significant contribution. Furthermore, individual interactions appear to be nonadditive (*i.e.*, cooperative).

### The aPKC kinase domain and PBM form the Par complex–binding surface for Par-3

**Figure 2:**
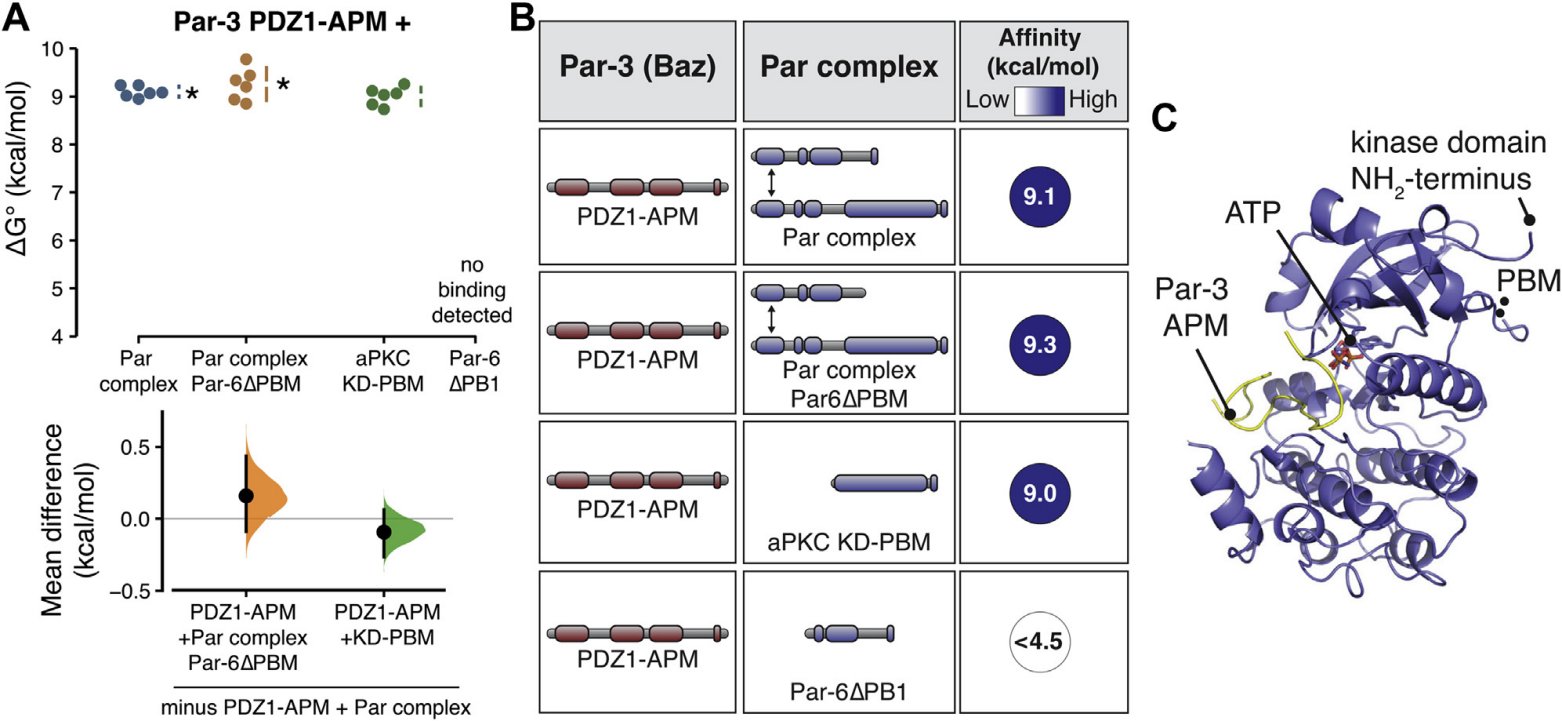
**The aPKC kinase domain and PDZ-binding motif form the Par complex binding site for Par-3.***A*, cumming estimation plot of Par-3–Par complex interaction energies measured using the supernatant depletion assay. Note: the data for PDZ1–APM binding to the Par complex is the same as shown in Figure 1. The *asterisk* indicates apparent value that may be the result of multiple binding interactions. *B*, the summary of binding energies for Par-3 interaction with the aPKC KD-PBM and Par complex lacking the Par-6 PBM. *C*, the structure of the aPKC kinase domain in complex with the Par-3 phosphorylation site (from PDB ID 5LI1; (20)) showing the relative position of the PBM and substrate binding sites. Note that electron density for the residues directly preceding the PBM, but not the PBM itself, is present in this structure. aPKC, atypical Protein Kinase C; APM, aPKC phosphorylation motif; PBM, PDZ-binding motif.

We sought to determine which interaction domains or motifs from the Par complex collaborate with the aPKC PBM to contribute binding energy for Par-3. Par-6 has been reported to contain a PBM that interacts with Par-3 PDZ1 or PDZ3 (9). When examining the effect of removing Par-6’s PBM on the overall interaction energetics, we were unable to detect a difference in the binding of Par-3 to the Par complex lacking the Par-6 PBM (Fig. 2, *A* and *B*; 9.3 kcal/mol; 8.9–9.6 95% CI). Given that our implementation of the supernatant depletion assay reliably detects binding energy differences on the order of 0.2 kcal/mol (*e.g.*, see confidence intervals in Fig. 1*C*) and that we were unable to detect an interaction between Par-3 PDZ1-APM and Par-6ΔPB1 (Fig. 2, *A* and *B*), we conclude that Par-3 interactions with the Par-6 PBM do not play a significant role in stabilizing Par-3 binding to the Par complex in the context of these purified components.

Given that the Par-6 PBM is not responsible for the additional interaction energy with Par-3, we sought to determine which Par complex interaction domains or motifs might contribute to the additional binding energy beyond the aPKC PBM. We found that the aPKC kinase domain along with the adjacent PBM (KD-PBM; Fig. 2*C*) fully recapitulated the interaction energy of the Par complex with Par-3 (Fig. 2, *A* and *B*; 9.0 kcal/mol; 8.8–9.2). Thus, in the context of these purified components, we do not find that the Par-3 PDZ1 interaction with the Par-6 PDZ or the PDZ1 and 3 interactions with the Par-6 PBM substantially contribute to the overall Par-3 and Par complex–binding energy.

### A conserved basic region NH_2_-terminal to Par-3 PDZ2 contributes to Par complex binding

**Figure 3:**
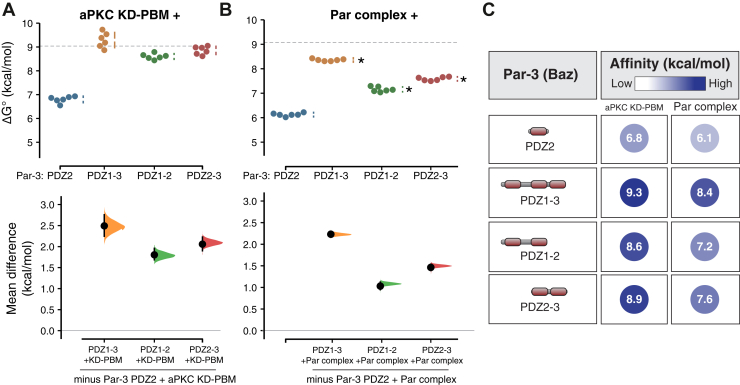
**Energetic contributions to the Par-3–Par complex interaction from the Par-3 PDZ domains.***A* and *B*, cumming estimation plots of Par-3–Par complex interaction energies measured using the supernatant depletion assay. The *dashed lines* indicate the binding energy of PDZ1-APM binding to the Par complex. *Asterisks* indicate apparent values that may be the result of multiple binding interactions. *C*, the summary of binding energies for Par-3 interaction with the aPKC KD-PBM and Par complex. aPKC, atypical Protein Kinase C; APM, aPKC phosphorylation motif; PBM, PDZ-binding motif.

We used both the Par complex and the isolated aPKC KD-PBM to identify which regions of Par-3 outside of PDZ2 contribute to the overall interaction energy. We found that a Par-3 fragment containing its three PDZ domains has a similar binding energy as PDZ1-APM (Fig. 3, *A*–*C*; 9.3 kcal/mol; 9.0–9.6 95% CI). This result indicates that Par-3’s phosphorylation site (APM) and the linker region connecting it to PDZ3 do not contribute significantly to the interaction. Note that ATP was present in our binding assay so that any interaction of the aPKC kinase domain with the APM was likely transient (and the interaction with the phosphorylated APM is weak) (10, 14). We did not detect any difference in the binding energy of Par-3 PDZ1-3 to the Par complex when ATP was replaced with ADP (Fig. S2*A*).

**Figure 4:**
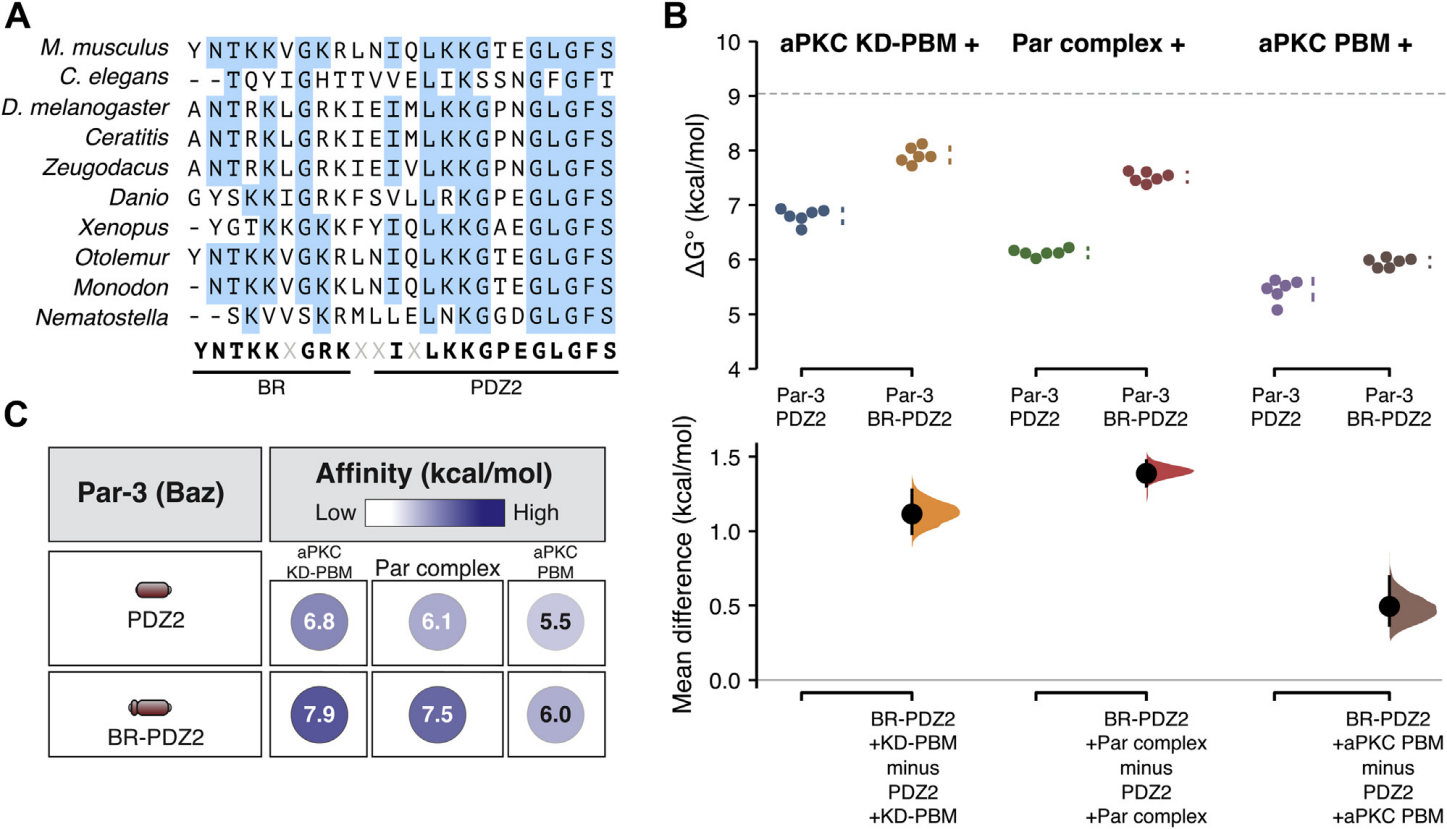
**A conserved BR contributes binding energy to the Par-3 PDZ2 interaction with the Par complex.***A*, a sequence alignment of the region NH2-terminal to the Par-3 PDZ2 from the Par-3 sequence from diverse animal species. *B*, cumming estimation plot of Par-3–Par complex interaction energies measured using the supernatant depletion assay. The *dashed lines* indicate the binding energy of PDZ1-APM binding to the Par complex. *C*, summary of binding energies for Par-3 PDZ2 and BR-PDZ2 interaction with the aPKC KD-PBM, Par complex, and aPKC PBM. aPKC, atypical Protein Kinase C; APM, aPKC phosphorylation motif; BR, basic region; PBM, PDZ-binding motif.

When examining the three Par-3 PDZ domains, we found that either PDZ1-2 or PDZ2-3 bound with binding energies similar to PDZ1-APM, although PDZ1-2’s was somewhat lower than PDZ2-3’s, an effect that was larger in the context of the full Par complex relative to the KD-PBM alone (Fig. 3). We noticed that an approximately 30 residue sequence NH_2_-terminal to the PDZ2 domain is enriched in basic amino acids and highly conserved in Par-3’s from diverse animal species (Fig. 4*A*). We termed this motif the basic region (BR) and found that including it with Par-3 PDZ2 (BR-PDZ2) significantly increased the binding energy of the interaction with the aPKC kinase domain and the full Par complex (Fig. 4, *B* and *C*). We conclude that the higher binding energy of PDZ1-2 than PDZ2 alone is contributed by the conserved BR motif.

### The Par-3 PDZ3 domain binds the aPKC kinase domain and PBM

**Figure 5:**
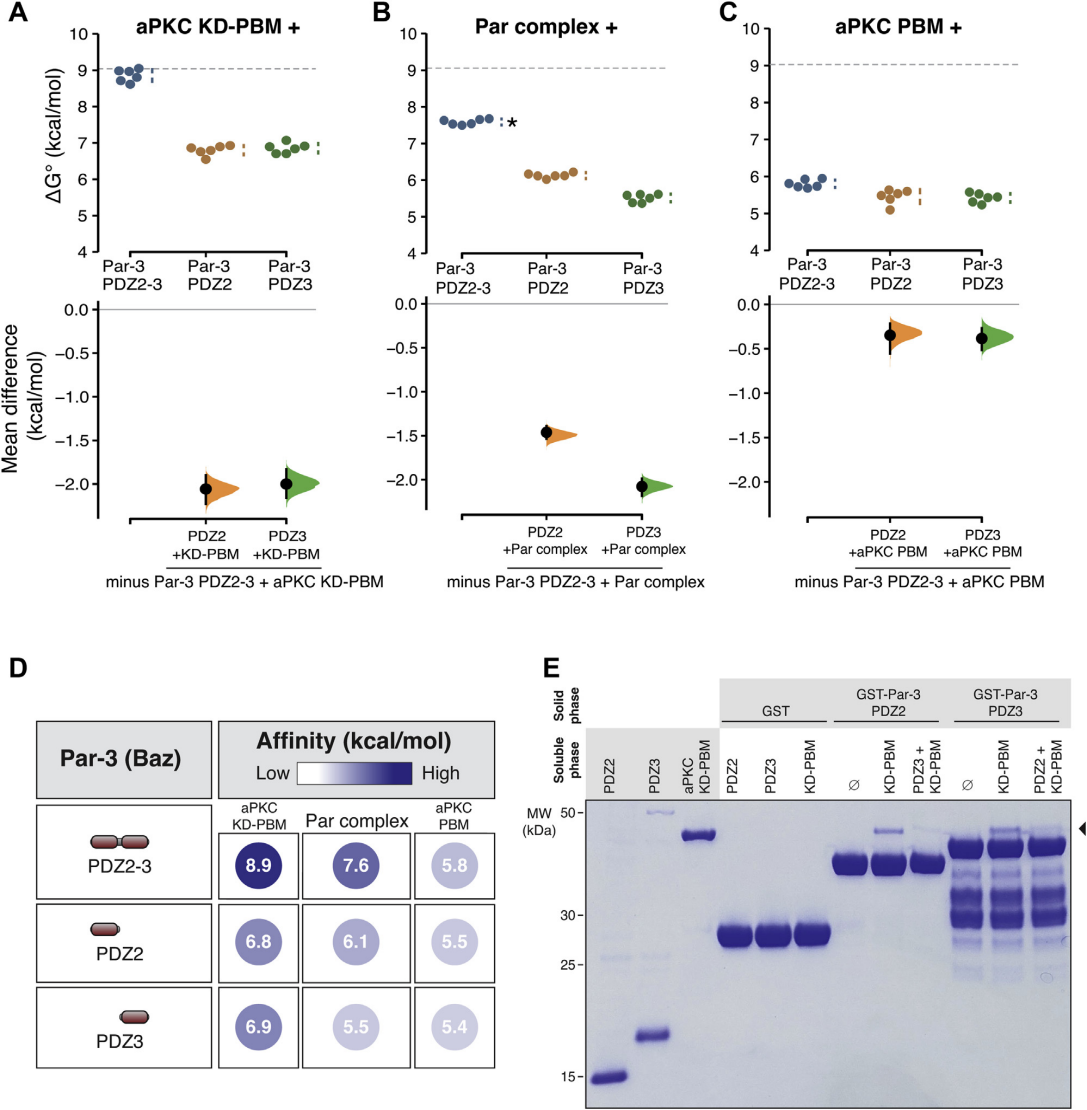
**Par-3 PDZ3 binds the aPKC kinase domain and PDZ binding motif.***A*–*C*, cumming estimation plots of Par-3 PDZ2 and PDZ3 interaction energies with the aPKC kinase domain–PBM (*A*), full Par complex (*B*), and aPKC PBM (*C*) measured using the supernatant depletion assay. The *dashed lines* represent the interaction energy of PDZ1-APM to the Par complex. The *asterisk* indicates apparent value that may be the result of multiple binding interactions. *D*, the summary of binding energies for Par-3 PDZ2 and PDZ3 interactions with the aPKC kinase domain-PBM, Par complex, and aPKC PBM. *E*, competition between Par-3 PDZ2 and PDZ3 for binding to aPKC KD-PBM. *Solid phase* (glutathione resin)–bound GST fused Par-3 PDZ2 or PDZ3 incubated with aPKC KD-PBM (*arrowhead*) and the indicated competing PDZ domain. *Shaded regions* of the legend indicate the fraction applied to the gel (soluble-phase or solid-phase components after mixing with soluble-phase components and washing). aPKC, atypical Protein Kinase C; APM, aPKC phosphorylation motif; PBM, PDZ-binding motif.

Like PDZ1-2, the combination of PDZ2 and 3 (Par-3 PDZ2-3) also bound aPKC KD-PBM and the full Par complex with higher affinity than PDZ2 alone (Fig. 5, *A*–*D*). In this case, the higher binding energy originates from PDZ3 as we discovered that it binds the aPKC KD-PBM with similar energy to PDZ2 and with somewhat less energy to the full Par complex (Fig. 5, *A*–*D*). We also found that PDZ3 binds the aPKC PBM with a similar energy as PDZ2. Like PDZ2, the binding energy of PDZ3 was higher for aPKC KD-PBM than the PBM alone.

Our results indicate that Par-3 PDZ2 and PDZ3 use a similar binding mode and therefore may compete for binding to aPKC KD-PBM. To test this hypothesis, we performed a competition experiment, first assembling a complex of the aPKC KD-PBM with PDZ2 and then adding PDZ3. We found that the presence of PDZ3 caused a significant decrease in the amount of aPKC bound to PDZ2 (Fig. 5*E*). Soluble PDZ2 was also able to displace PDZ3 from aPKC KD-PBM (Fig. 5*E*). The competitive binding for the two PDZ domains suggests that PDZ2 and PDZ3 each binds a distinct Par complex. Furthermore, the increased binding affinity when both PDZ2 and 3 are present (*e.g.*, PDZ2-3) relative to the individual domains likely arises from an avidity effect in which more than one Par complex is participating in the interaction.

### Par-3 BR-PDZ2-3 binding to aPKC KD-PBM recapitulates the overall interaction energy

**Figure 6:**
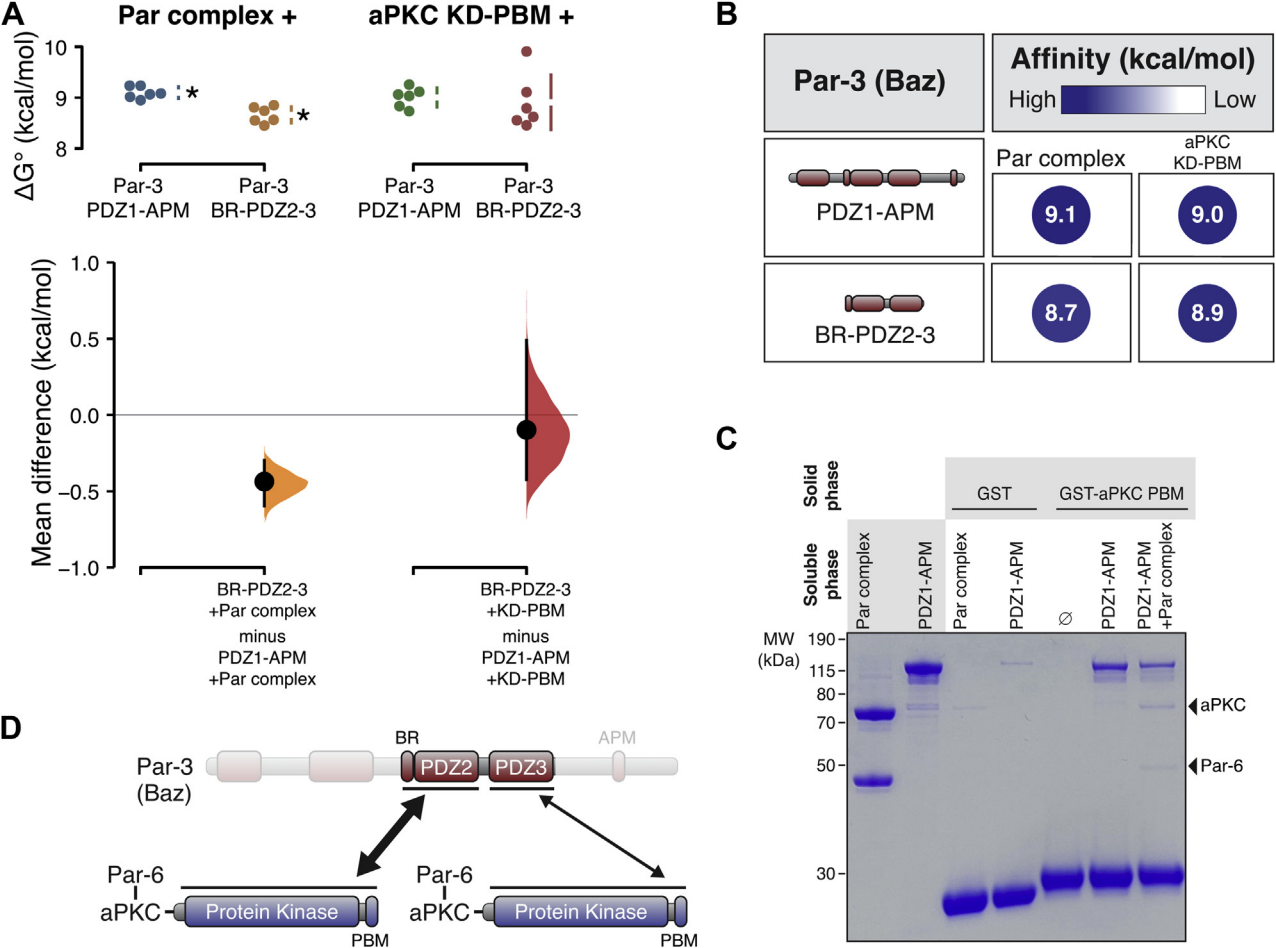
**Par-3 BR-PDZ2-3 binding to the aPKC kinase domain-PBM fully recapitulates the Par-3 interaction with the Par complex.***A*, cumming estimation plot of Par-3 BR-PDZ2-3 interaction energies with the full Par complex and aPKC KD-PBM measured using the supernatant depletion assay. *Asterisks* indicate apparent values that may be the result of multiple binding interactions. *B*, summary of binding energies for BR-PDZ2-3 interaction with the full Par complex and aPKC KD-PBM. *C*, Par-3 PDZ1-APM can bind the Par complex while binding to the aPKC PBM. *D*, the model for the interaction of Par-3 with the Par complex. aPKC, atypical Protein Kinase C; APM, aPKC phosphorylation motif; BR, basic region; PBM, PDZ-binding motif.

Taken together, our results suggest that the binding energy of the Par-3 interaction with the Par complex arises from separate interactions of the BR-PDZ2 and PDZ3 with the aPKC KD-PBM. As shown in Figure 6, *A* and *B*, Par-3 BR-PDZ2-3 nearly completely recapitulates the binding energy of PDZ1-APM. To determine if distinct Par complexes can bind to PDZ2 and PDZ3, we asked whether the Par-3 PDZ1-APM adsorbed to the solid phase *via* the aPKC PBM could recruit the Par complex. We found that the Par complex was specifically adsorbed to GST-aPKC PBM in the presence of Par-3 PDZ1-APM (Fig. 6*C*). We conclude that distinct interactions of BR-PDZ2 and PDZ3 with aPKC KD-PBM form the basis of the Par-3 interaction with the Par complex (Fig. 6*D*). In the context of these purified proteins, we do not detect a significant contribution from the interaction of PDZ1 or PDZ3 with the Par-6 PBM, the interaction of PDZ1 with the Par-6 PDZ, or the interaction of the aPKC kinase domain with its phosphorylation site.

## Discussion

The nature of the Par-3 interaction with the Par complex has been enigmatic (15, 16, 17, 18, 19). In this study, we used a quantitative biochemical approach with purified, full-length Par complex and a region of Par-3 that contains all known binding motifs to address the challenge of understanding this complicated interaction. We found that Par-3 PDZ2 and PDZ3 binding to the aPKC KD-PBM nearly fully recapitulates the binding energy of the overall interaction between Par-3 and the Par complex. We note that these interactions most closely resemble the previously identified interaction of Par-3 PDZ2-3 with full-length aPKC using a yeast two-hybrid assay (8). We used the *Drosophila* versions of these proteins and, while the Par complex is highly conserved, it is possible that the proteins from other organisms behave differently. Here, we examine the implications of our quantitative findings on Par-3’s role in Par-mediated polarity.

We used binding energy to evaluate the relative contribution of each of the identified Par-3 interactions with the Par complex. The binding energies of several of the interactions in the context of isolated Par complex fragments have been previously reported. The interaction of the aPKC kinase domain with the Par-3 APM has been reported to be very high (8.6 kcal/mol) (20). However, this interaction was measured in the absence of ATP, conditions which prevent substrate turnover and are consequently not physiologically relevant (10, 14). We did not detect any contribution to the overall interaction between Par-3 and the Par complex from the Par-3 APM when ATP was present. The interactions of Par-3 PDZ1 and PDZ3 with the Par-6 PBM were measured using NMR and were found to be weak (5.0 and 5.8 kcal/mol, respectively). While these affinities are low, they are above the limit of detection of the supernatant depletion assay. However, we did not detect any significant contributions from the Par-6 PBM in the context of the Par complex binding to Par-3; we did not detect an interaction of Par-3 with Par-6/aPKCΔPBM nor did we detect a change in affinity when the Par-6 PBM was removed (*i.e.*, Par-6ΔPBM/aPKC).

An analysis of the Par-3 domains required for polarity in the *Caenorhabditis elegans* zygote found that PDZ1 and 3 were dispensable but the oligomerization domain and PDZ2 were necessary (21). A similar analysis found that the interaction of the Par-6 PDZ domain with Par-3 was also dispensable for the Par complex function (11). An examination of the Par-6 PBM found that it is not required for viability in *Drosophila* and its removal did not have a measurable effect on Par-6 recruitment to the cortex of the embryonic epithelium except when the Par-6 PDZ was also removed (9). In a study of aPKC PBM function, aPKCΔPBM was not polarized to the apical membrane during the asymmetric division of *Drosophila* larval neural stem cells (10). These functional results are consistent with the primacy of the aPKC PBM in binding to Par-3. They also suggest that the biochemical redundancy between Par-3 PDZ2 and PDZ3 does not translate to *in vivo* function, either because of the lower affinity of PDZ3 or because PDZ2 participates in other essential functions besides binding to the Par complex.

Our results indicate that the aPKC kinase domain participates in the interaction with Par-3 PDZ2 and PDZ3. The nature of this interaction is not known, but the proximity of the aPKC PBM to the kinase domain is suggestive (Fig. 2*C*) (20). Binding to the PBM would bring PDZ surfaces outside of the PBM pocket near the kinase domain and could lead to the so-called “docking” interactions that occur between protein kinase substrates and regions away for the kinase domain active site (22). Another interesting feature of the binding energetics results is the higher binding energy of the Par-3 PDZ domains to the aPKC KD-PBM than the full Par complex (Fig. 5*A versus* Fig. 5*B*). The lower binding affinity to the full Par complex suggests that autoregulation may be present in the system. Future efforts will be directed at exploring the nature of these interactions and any role they may have in regulating aPKC activity.

## Experimental procedures

### Cloning

GST-, MBP-, and his-tagged Par-3 constructs, GST-aPKC PBM, and his aPKC kinase domain-PBM (residues 259–606) were cloned as previously described (10) using Gibson cloning (New England Biolabs), Q5 mutagenesis (New England Biolabs), or traditional methods. In addition to an N-terminal MBP tag, the Par-3 PDZ1-APM (residues 309–987) construct also contained a C-terminal his-tag. Par complex components (aPKC and his-Par-6) were cloned into pCMV (human cytomegalovirus) as previously described (10, 23). Please see the Key resources table for additional information on specific constructs.

### Expression

All proteins, except for Par complex constructs, were expressed in *Escherichia coli* (strain BL21 DE3). Constructs were transformed into BL21 cells, grown overnight at 37 °C on LB+ampicillin (Amp; 100 μg/ml). Resulting colonies were selected and used to inoculate 100 ml LB+Amp starter cultures. Cultures were grown at 37 °C to an *A*_600_ of 0.6 to 1.0 and then diluted into 2 l LB+Amp cultures. At an *A*_600_ of 0.8 to 1.0, expression was induced with 0.5 mM IPTG for 2 to 3 h. Cultures were centrifuged at 4400*g* for 15 min to pellet cells. The media was removed and pellets were resuspended in nickel lysis buffer [50 mM NaH_2_PO_4_, 300 mM NaCl, 10 mM Imidazole, pH 8.0], GST lysis buffer [1XPBS, 1 mM DTT, pH 7.5] or maltose lysis buffer [20 mM Tris, 200 mM NaCl, 1 mM EDTA, 1 mM DTT, and pH 7.5], as appropriate. Resuspended pellets were frozen in liquid N_2_ and stored at −80 °C.

Par complex constructs were expressed in HEK 293F cells (Thermo Fisher Scientific), as previously described (10, 23). Briefly, cells were grown in FreeStyle 293 expression media (Thermo Fisher Scientific) in shaker flasks at 37 °C with 8% CO_2_. Cells were transfected with 293fectin (Thermo Fisher Scientific) or ExpiFectamine (Thermo Fisher Scientific), according to the manufacturer’s protocol. After 48 h, cells were collected by centrifugation (500*g* for 5 min). Cell pellets were resuspended in nickel lysis buffer, frozen in liquid N_2_, and stored at −80 °C.

### Purification

Resuspended *E. coli* pellets were thawed and cells were lysed by probe sonication using a Sonic Dismembrator (Model 500, Thermo Fisher Scientific; 70% amplitude, 0.3/0.7 s on/off pulse, 3 × 1 min). 293F cell pellets were lysed similarly using a microtip probe (70% amplitude, 0.3/0.7 s on/off pulse, 4 × 1 min). Lysates were centrifuged at 27,000*g* for 20 min to pellet cellular debris. GST- and MBP-tagged protein lysates were aliquoted, frozen in liquid N_2_, and stored at −80 °C.

His-tagged protein lysates, except aPKC KD-PBM, were incubated with HisPur Ni-NTA (Thermo Fisher Scientific) or HisPur Cobalt (Thermo Fisher Scientific) resin for 30 min at 4 °C and then washed 3× with nickel lysis buffer. For 293F lysates, 100 μM ATP and 5 mM MgCl_2_ were added to the first and second washes. Proteins were eluted in 0.5 to 1.5 ml fractions with nickel elution buffer (50 mM NaH_2_PO_4_, 300 mM NaCl, 300 mM Imidazole, pH 8.0). For all proteins, aside from the Par complex, fractions containing protein were pooled, buffered exchanged into 20 mM Hepes with pH 7.5, 100 mM NaCl, and 1 mM DTT using a PD10 desalting column (Cytiva), concentrated using a Vivaspin20 protein concentrator spin column (Cytiva), aliquoted, frozen in liquid N_2_, and stored at −80 °C. For the Par complex, proteins were further purified using anion exchange chromatography on an AKTA FPLC protein purification system (Amersham Biosciences). Following his-purification, fractions were pooled, buffered, and exchanged into 20 mM Hepes pH 7.5, 100 mM NaCl, 1 mM DTT, 100 μM ATP, and 5 mM MgCl_2_ using a PD10 desalting column (Cytiva). Buffer-shifted protein was injected onto a Source Q (Cytiva) column and eluted over a salt gradient of 100 to 550 mM NaCl. Fractions containing the Par complex were pooled, buffered, exchanged into 20 mM Hepes pH 7.5, 100 mM NaCl, 1 mM DTT, 100 μM ATP, and 5 mM MgCl_2_ using a PD10 desalting column (Cytiva), concentrated using a Vivaspin20 protein concentrator spin column (Cytiva), aliquoted, frozen in liquid N_2_, and stored at −80 °C.

Due to solubility issues, aPKC KD-PBM was expressed in *E. coli* and his-purified partially under denaturing conditions. Following sonication and centrifugation (described previously), the soluble fraction was discarded and the insoluble pellet was resuspended in 50 mM NaH_2_PO_4_, 300 mM NaCl, 10 mM Imidazole, 8 M Urea pH 8.0. Centrifugation was repeated (27,000*g* for 20 min) and the resulting soluble phase was incubated with HisPur Ni-NTA resin (Thermo Fisher Scientific) for 30 min at 4 °C. Resin was washed and eluted as described previously. Purified protein was aliquoted, frozen in liquid N_2_, and stored at −80 °C.

### Quantitative binding assay

For all solid phase proteins, except Par-3 PDZ1-APM and PDZ1-APMΔPDZ2, GST lysates were incubated with glutathione agarose resin (Gold Biotechnology; 50 μl resin per 0.5–1.5 ml of lysate) for 30 min at 4 °C and then washed 6× (3× quick washes, followed by 3 × 5 min washes at room temp) with binding buffer (10 mM Hepes pH 7.5, 100 mM NaCl, 1 mM DTT 200 μM ATP, 5 mM MgCl_2_ and 0.1% Tween-20). After washing, the resin was resuspended in 50 μl binding buffer to create a 50% slurry. Par-3 PDZ1-APM and PDZ1-APMΔPDZ2 were double tagged (N-terminal MBP-tag and C-terminal his-tag). Par-3 PDZ1-APM and PDZ1-APMΔPDZ2 were first his-purified (as described previously) before incubation with amylose resin (NEB). Amylose-bound Par-3 was then washed and resuspended in binding buffer as described for GST proteins.

Separately, unlabeled resin (amylose or glutathione resin, as appropriate) was washed 3× and resuspended in a 50% slurry with binding buffer. GST- or MBP-labeled resin was then serially diluted 1:1 (30 μl of 50% slurry) with unlabeled resin to create a gradient of the GST/MBP-tagged protein. Unlabeled resin was used as a negative control for binding. Soluble protein (“receptor”) was added to the solid phase protein (“ligand”) and incubated for 1 h at 20 °C with rotational mixing (see Table S1 for the solid phase-soluble phase combination for each experiment), except for MBP-Par-3 constructs. Due to high levels of leaching into the supernatant from amylose-bound MBP-tagged proteins, MBP–Par-3 assays were incubated for 10 min (we confirmed that GST-Par-3 PDZ1-3 incubated for 1 h produced indistinguishable results to MBP-Par-3 PDZ1-3 incubated for 10 min; Fig. S2*B*).

Following incubation, a sample of the supernatant was removed from each tube and combined with 4× LDS sample buffer (Thermo Fisher Scientific). Samples were run on a Bis-Tris gel, stained with Coomassie Brilliant Blue R-250 ( Gold Biotechnology) and band intensity was quantified using ImageJ (v1.53a). The fraction of R (soluble phase) bound to L (solid phase) at a specific concentration of L ([*L*] = *x*) was determined using the following equation:

$$Fraction\;bound\left(\left[L\right]=x\right)=1-\frac{I_{\left[L\right]=x}}{I_{\left[L\right]=0}}$$

where *I*_[*L*] = *x*_ represents the intensity of the receptor (soluble phase) band at ligand (solid phase) concentration “*x*” following equilibration, and *I*_[*L*] = 0_ is the receptor band intensity without any ligand present. The dilution of the solid phase that resulted in 30 to 60% depletion (F_b_ = 0.3–0.6) was determined using a ligand titration, and the assay was repeated in sextuplicate at this dilution. The solid phase concentration ([*L*]) was determined by gel analysis using a standard protein of known concentration.

The binding equilibrium dissociation constant (*K*_*d*_) was evaluated from the fraction bound (F_b_) using the single site binding equation derived below:

$$K_{d}=\;\frac{\left[L\right]\left[R\right]}{\left[LR\right]}$$

where, [*L*], [*R*] represents concentration of free ligand (solid phase) and receptor (soluble phase) at equilibrium and [*LR*] represents concentration of complex at equilibrium.

$$K_{d}=\frac{\left({\left[L\right]}_{total}-\left[LR\right]\right)\left({\left[R\right]}_{total}-\left[LR\right]\right)}{\left[LR\right]}$$

$$K_{d}=\frac{\left({\left[L\right]}_{total}-F_{b}{\left[R\right]}_{total}\right)\left({\left[R\right]}_{total}-F_{b}{\left[R\right]}_{total}\right)}{F_{b}{\left[R\right]}_{total}}$$

The binding energy was calculated using the equation for the standard Gibbs free energy change:

$$\Delta \text{G}{}^{\circ}=-\text{RT ln}\left[K_{d}\right]$$

Binding results from experiments using Par-3 variants that could potentially bind more than one Par complex are labeled as “apparent” to emphasize that the binding energy could arise from multiple interactions.

The data was visualized and analyzed using Excel (v16.53), GraphPad Prism (v9.2, https://www.graphpad.com/scientific-software/prism/), and the DABEST (https://github.com/ACCLAB/DABEST-python) (24) software packages. Confidence intervals were estimated using the bootstrap method as implemented in DABEST.

### Qualitative binding assays

For GST pulldown assays, GST lysates were incubated with glutathione agarose resin (Gold Biotechnology) for 30 min at 4 °C and then washed 6× (3× quick washes, followed by 3 × 5 min washes at room temp) with binding buffer (10 mM Hepes pH 7.5, 100 mM NaCl, 1 mM DTT 200 μM ATP, 5 mM MgCl_2_ and 0.1% Tween-20). Soluble proteins were added to GST-bound proteins, as indicated, and incubated at room temperature with rotational agitation for 30 to 60 min. Resin was then washed 3× with binding buffer and protein was eluted with 4× LDS sample buffer (Thermo Fisher Scientific). Samples were run on a Bis-Tris gel and stained with Coomassie Brilliant Blue R-250 (Gold Biotechnology).

### Key resources table

**Table:** 

Reagent type (species) or resource	Designation	Source or reference	Identifiers	Additional information
Recombinant protein	Par complex (his-Par-6, aPKC)	PMID: 32084408		expressed in 293F cells from pCMV his-Par-6 1–351 and pCMV aPKC 1–606
Recombinant protein	Par complex aPKCΔPBM	PMID: 32084408		expressed in 293F cells from pCMV his-Par-6 1–351 and pCMV aPKC 1–600
Recombinant protein	Par complex Par-6ΔPBM	PMID: 32084408		expressed in 293F cells from pCMV his-Par-6 1–343 and pCMV aPKC 1–606
Recombinant protein	GST-aPKC PBM	PMID: 32084408		expressed in BL21 cells from pGEX aPKC 583–606
Recombinant protein	GST-Par-3 PDZ2-3	This article		Cloned by Q5 mutagenesis; expressed in BL21 cells from pGEX Par-3 444–741
Recombinant protein	GST-Par-3 PDZ1-2	This article		Gibson cloning; expressed in BL21 cells from pGEX Par-3 309–533
Recombinant protein	GST-Par-3 PDZ1-3	This article		Gibson cloning; expressed in BL21 cells from pGEX Par-3 309–741
Recombinant protein	GST-Par-3 BR-PDZ2	This article		Gibson cloning; expressed in BL21 cells from pGEX Par-3 426–533
Recombinant protein	GST-Par-3 PDZ2	This article		Gibson cloning; expressed in BL21 cells from pGEX Par-3 444–533
Recombinant protein	GST-Par-3 BR-PDZ2-3	This article		Gibson cloning; expressed in BL21 cells from pGEX Par-3 426–741
Recombinant protein	GST-Par-3 PDZ3	This article		Gibson cloning; expressed in BL21 cells from pGEX Par-3 616–741
Recombinant protein	aPKC KD-PBM	This article		Cloned using traditional methods; expressed in BL21 cells from pBH aPKC 259–606; his-purified under denaturing conditions
Recombinant protein	Par-3 PDZ2	This article		Gibson cloning; expressed in BL21 cells from pET19 Par-3 444–533; his-purified
Recombinant protein	Par-3 BR-PDZ2	This article		Gibson cloning; expressed in BL21 cells from pET19 Par-3 426–533; his-purified
Recombinant protein	Par-3 PDZ2-3	This article		Cloned by Q5 mutagenesis; expressed in BL21 cells from pET19 Par-3 444–741; his-purified
Recombinant protein	Par-3 PDZ3	This article		Gibson cloning; expressed in BL21 cells from pET19 Par-3 616–741; his-purified
Recombinant protein	MBP-Par-3 PDZ1-APM	PMID: 32084408		expressed in BL21 cells from pMAL Par-3 309–987-his; C-terminal his-tag; his-purified prior to use in binding assay
Recombinant protein	MBP-Par-3 PDZ1-APMΔPDZ2	PMID: 32084408		expressed in BL21 cells from pMAL Par-3 309–987Δ437–533-his; C-terminal his-tag; his-purified prior to use in binding assay
Recombinant DNA reagent	pCMV (mammalian expression plasmid)	Thermo Fisher Scientific	10586014	
Recombinant DNA reagent	pMal C4X (bacterial expression plasmid)	Addgene	75288	
Recombinant DNA reagent	pGEX 4Ti (bacterial expression plasmid)	Amersham	27458001	
Recombinant DNA reagent	pBH (bacterial expression plasmid)	PMID: 15023337		
Recombinant DNA reagent	pET19 (bacterial expression plasmid)	Millipore Sigma (Novagen)	69677	
Bacterial strain	BL21-DE3			used for recombinant protein expression
Bacterial strain	TG1			used for DNA cloning
Cell line (human)	FreeStyle 293-F	Thermo Fisher Scientific	R79007	
Chemical	IPTG	Gold Biotechnology	I2481C100	used at 0.5 mM
Chemical	293fectin	Thermo Fisher Scientific	12347019	
Chemical	ExpiFectamine 293 Transfection Kit	Thermo Fisher Scientific	A14524	
Chemical	Freestyle 293 expression Medium	Thermo Fisher Scientific	12338018	
Chemical	Opti-Mem	Thermo Fisher Scientific	3198588	
Chemical	HisPur cobalt resin	Thermo Fisher Scientific	89965	
Chemical	HisPur Ni-NTA resin	Thermo Fisher Scientific	88222	
Chemical	Amylose Resin	NEB	E8021L	
Chemical	Glutathione Resin	Gold Biotechnology	G250-100	
Chemical	Source Q anion exchange resin	GE Healthcare	17-1275-01	
Chemical	LB Broth, Miller	Millipore Sigma	71753-6	
Chemical	4× BOLT LDS sample buffer	Thermo Fisher Scientific	B0007	
Chemical	20× BOLT MES SDS running buffer	Thermo Fisher Scientific	B0002	
Chemical	Coomassie Brilliant Blue R-250	Gold Biotechnology	C-461-5	
Commercial kit	Q5 Site-Directed Mutagenesis Kit	NEB	E0552S	
Commercial kit	Gibson Assembly Cloning Kit	NEB	E5510S	
Other	Bolt 12% Bis-Tris Gels	Thermo Fisher Scientific	NW00125BOX	
Other	PD10 Desalting columns		95017-001	
Other	VivaSpin 20 sample concentrators MWCO 30kD	Cytiva	28932361	
Other	VivaSpin 20 sample concentrators MWCO 10kD	Cytiva	28932360	
Other	VivaSpin 20 sample concentrators MWCO 5kD	Cytiva	28932359	
Other	Shaker Flasks – 125 ml	VWR	89095-258	
Other	Shaker Flasks – 250 ml	VWR	89095-266	
Software	ImageJ	NIH		v1.53a; https://imagej.nih.gov/ij/
Software	GraphPad Prism	GraphPad Software		v.9.2
Software	Estimation Statistics BETA	PMID: 31217592		www.estimationstats.com

## Data availability

All processed ΔG° values are available within the article. Raw data used to calculate fraction bound are available on request to K. E. P.

## Supporting information

This article contains supporting information.

## Conflict of interest

The authors declare that they have no conflicts of interest with the contents of this article.
