# Supplementary material for: Energetic determinants of animal cell polarity regulator Par-3 interaction with the Par complex
Source: J Biol Chem. 2022 Jul 1;298(8):102223. doi: 10.1016/j.jbc.2022.102223 (PMC9352551; doi:10.1016/j.jbc.2022.102223)
Supplement: Supplemental Table S1 [file mmc1.docx]

Supplemental Table 1

|  |  |  | **ΔG° (kcal/mol)** | **K_d_ (uM)** |
| --- | --- | --- | --- | --- |
|  | **Solid phase** | **Soluble phase** | **Mean  [95% CI]** | **Mean  [95% CI]** |
| **PDZ1-APM +Par complex** | MBP-Par-3  309-987-his | aPKC 1-606  his-Par6 1-351 | 9.10 [8.98-9.22] | 0.17 [0.13-0.20] |
| **PDZ2 +aPKC PBM** | GST-aPKC  583-606 | his-Par-3  444-533 | 5.46  [5.25-5.66] | 89.8  [52.4-127.1] |
| **PDZ1-APM +Par complex aPKCΔPBM** | MBP-Par-3  309-987-his | aPKC 1-600  his-Par6 1-351 | No binding detected | No binding detected |
| **PDZ1-APM ΔPDZ2 +Par complex** | MBP-Par-3  309-987  Δ437-533 | aPKC 1-606  his-Par6 1-351 | 5.61  [5.56-5.66] | 65.4  [59.5-71.2] |
| **PDZ1-APM +aPKC PBM** | GST-aPKC  583-606 | MBP-Par-3  309-987-his | 5.47  [5.37-5.56] | 84.5  [71.8-97.3] |
| **PDZ1-APM +Par complex Par6ΔPBM** | MBP-Par-3  309-987-his | aPKC 1-606  his-Par6 1-343 | 9.26  [8.90-9.62] | 0.14  [0.06-0.22] |
| **PDZ1-APM +KD-PBM** | MBP-Par-3  309-987-his | his-aPKC  259-606 | 9.01  [8.81-9.21] | 0.20  [0.13-0.27] |
| **PDZ2 +KD-PBM** | GST-Par-3  444-533 | his-aPKC  259-606 | 6.80  [6.65-6.94] | 8.72 [6.30-11.14] |
| **PDZ1-3 +KD-PBM** | GST-Par-3  309-741 | his-aPKC  259-606 | 9.29  [8.96-9.62] | 0.13  [0.06-0.21] |
| **PDZ1-2 +KD-PBM** | GST-Par-3  309-533 | his-aPKC  259-606 | 8.60  [8.48-8.73] | 0.39  [0.31-0.47] |
| **PDZ2-3 +KD-PBM** | GST-Par-3  444-741 | his-aPKC  259-606 | 8.86  [8.67- 9.04] | 0.26  [0.18- 0.34] |
| **PDZ2 +Par complex** | GST-Par-3  444-533 | aPKC 1-606  his-Par6 1-351 | 6.13  [6.06-6.20] | 27.09  [23.79-30.38] |
| **PDZ1-3 +Par complex** | GST-Par-3  309-741 | aPKC 1-606  his-Par6 1-351 | 8.36  [8.31-8.40] | 0.59  [0.54-0.63] |
| **PDZ1-2 +Par complex** | GST-Par-3  309-533 | aPKC 1-606  his-Par6 1-351 | 7.16  [7.05-7.27] | 4.65  [3.81-5.48] |
| **PDZ2-3 +Par complex** | GST-Par-3  444-741 | aPKC 1-606  his-Par6 1-351 | 7.59  [7.51-7.67] | 2.20  [1.90-2.50] |
| **BR-PDZ2 +KD-PBM** | GST-Par-3  426-533 | his-aPKC  259-606 | 7.91  [7.76-8.07] | 1.28  [0.95-1.62] |
| **BR-PDZ2 +Par complex** | GST-Par-3  426-533 | aPKC 1-606  his-Par6 1-351 | 7.51  [7.41-7.61] | 2.52  [2.08-2.95] |
| **BR-PDZ2 +aPKC PBM** | GST-aPKC  583-606 | his-Par-3  426-533 | 5.95  [5.86-6.04] | 36.69  [31.02-42.37] |
| **PDZ3 +KD-PBM** | GST-Par-3  616-741 | his-aPKC  259-606 | 6.85  [6.71-7.00] | 7.89  [5.97-9.82] |
| **PDZ3 +Par complex** | GST-Par-3  616-741 | aPKC 1-606  his-Par6 1-351 | 5.51  [5.39-5.63] | 78.85  [62.31-95.38] |
| **PDZ3 +aPKC PBM** | GST-aPKC  583-606 | his-Par-3  616-741 | 5.42  [5.29-5.56] | 92.46  [70.47-114.45] |
| **PDZ2-3 +aPKC PBM** | GST-aPKC  583-606 | his-Par-3  444-741 | 5.80  [5.69-5.92] | 47.56  [38.47-56.65] |
| **BR-PDZ2-3 +KD-PBM** | GST-Par-3  426-741 | his-aPKC  259-606 | 8.91  [8.34-9.48] | 0.29  [0.12-0.47] |
| **BR-PDZ2-3 +Par complex** | GST-Par-3  426-741 | aPKC 1-606  his-Par6 1-351 | 8.66  [8.49-8.83] | 0.36  [0.25-0.46] |
