# Supplementary material for: Energetic determinants of animal cell polarity regulator Par-3 interaction with the Par complex
Source: J Biol Chem. 2022 Jul 1;298(8):102223. doi: 10.1016/j.jbc.2022.102223 (PMC9352551; doi:10.1016/j.jbc.2022.102223)
Supplement: Supplemental Figures S1 and S2 [file mmc2.pdf]

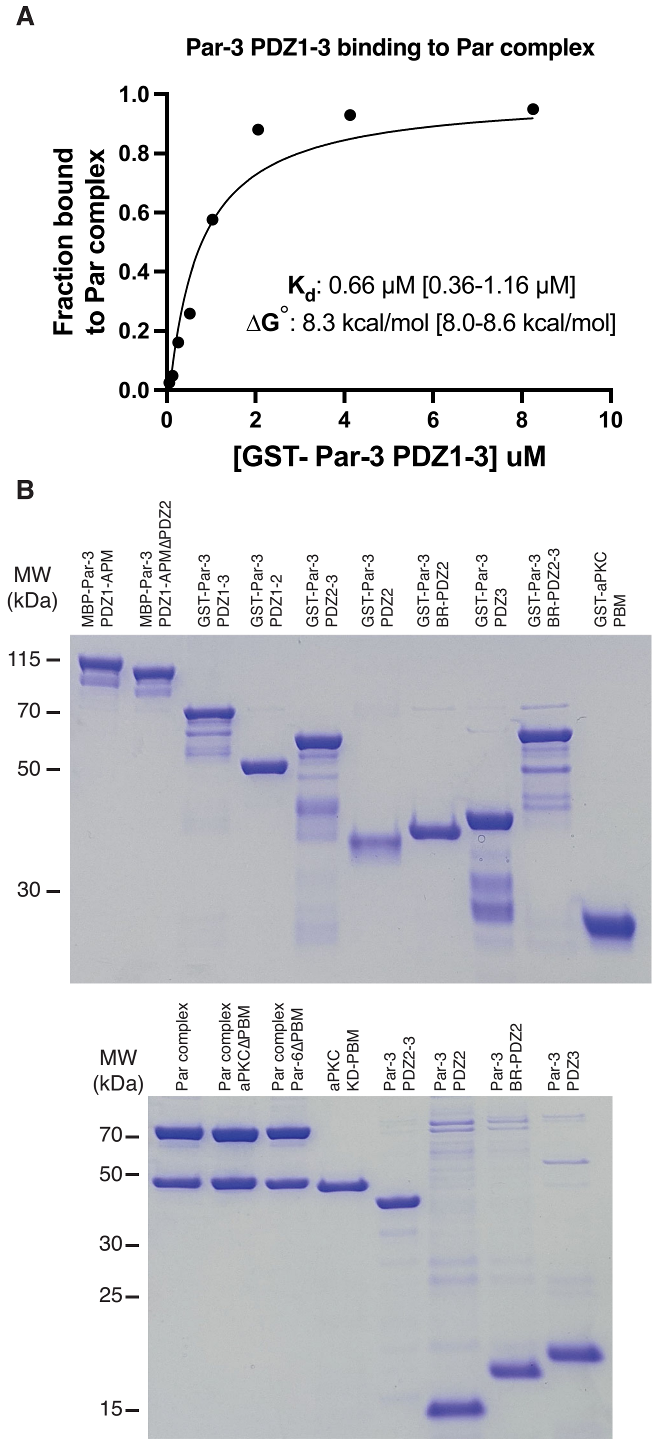

Figure S1 Supernatant depletion binding assay. (**A**) Dose response curve for binding of Par-3 PDZ1-3 to the Par complex. 95% confidence intervals are shown for best fit values. (**B**) Protein reagents used in this study.

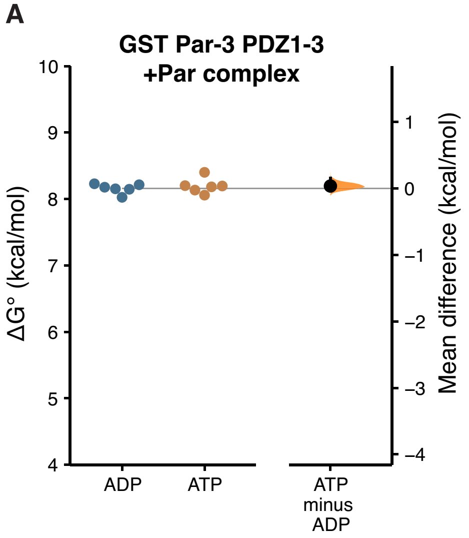

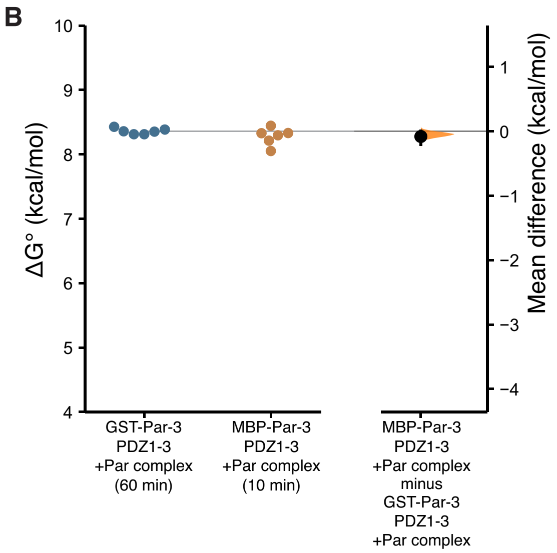

Figure S2 Supernatant depletion binding assay. (**A**) Effect of nucleotide state on binding of Par-3 PDZ1-3 to the Par complex. (**B**) Time dependence of binding energy measurement for Par-3 PDZ1-3 binding to the Par complex .
